# Symmetry Breaking in a Triferrous Extended Metal Atom Chain

**DOI:** 10.1021/acs.inorgchem.4c02752

**Published:** 2024-10-10

**Authors:** Jefferson E. Bates, Jack N. McKeon, Gary L. Guillet

**Affiliations:** † Department of Chemistry & Fermentation Sciences, Appalachian State University, Boone, North Carolina 28608-2021, United States; ‡ Department of Chemistry, Furman University, Greenville, South Carolina 29613, United States

## Abstract

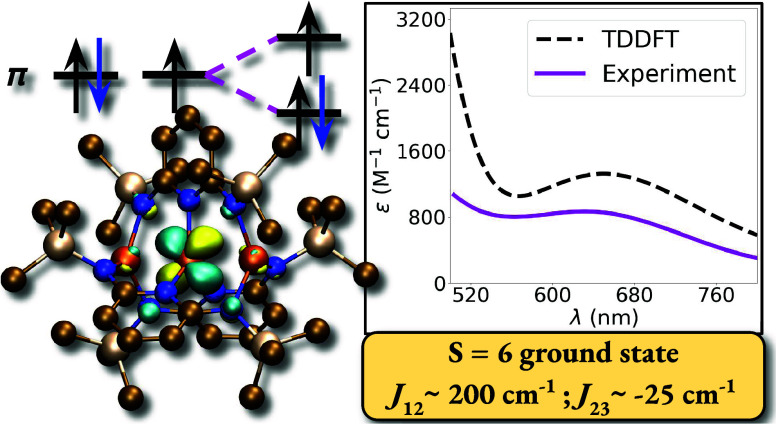

Semilocal and random phase approximation (RPA) density functional theory (DFT) and complete active space (CASSCF + NEVPT2) methodologies were applied to investigate a new class of extended metal atom chain (EMAC) complexes. A novel triferrous complex has been synthesized recently that does not utilize the usual 2,2′-dipyridylamine (dpa) ligand framework, which essentially always results in a tetragonal coordination environment and general formula M_3_(dpa)_4_X_2_, where X is an anion. Instead, the triferrous complex utilizes a dianionic, 2,6-bis(trimethylsilylamido)pyridine ligand (L^2–^) resulting in the formation of trigonal complexes with general formula **Fe**_**3**_**L**_**3**_. To better understand the electronic structure of this complex, calculations were utilized to explore the experimentally isolated **Fe**_**3**_**L**_**3**_, and a smaller theoretical complex, in order to compare and contrast with the traditional dpa-based EMACs. Due to the absence of anionic, axial ligands, the sigma nonbonding orbitals formed from the metal d orbitals are lower in energy than in the dpa complexes, and compete with the pi bonding orbitals for occupation in the **Fe**_**3**_**L**_**3**_ complex. While the idealized geometry of these complexes is *D*_3*h*_, a helical distortion of the ligands and subsequent electronic symmetry breaking due to Jahn–Teller distortions are predicted utilizing both semilocal and RPA DFT methods, ending in a *C*_2_ structure that closely matches the reported crystal structure. Predicted Mössbauer isomer shifts and ultraviolet/visible (UV/vis) spectra also agree with the experimental data available in the literature. Magnetic coupling constants also indicate ferromagnetic coupling between nearest neighbor irons. Two-dimensional (2D) potential energy surfaces were calculated for a range of fixed Fe–Fe bond lengths, revealing a flat potential energy surface over a wide range of Fe–Fe bond lengths and verifying the ability of RPA to act as a higher-level check on semilocal DFT results. In order to verify the predicted high-spin ground state, CASSCF + NEVPT2 was applied to selected molecular configurations and confirmed the predictions made by DFT. These calculations shed light on the physical ground state electron configuration of **Fe**_**3**_**L**_**3**_ and correlate this electronic configuration with the available experimental data.

## Abstract 2

Utilizing density functional theory and complete active space calculations, the nature of the high-spin ground state for a novel triferrous extended metal atom chain is thoroughly explored. The high-symmetry state of this complex is susceptible to Jahn−Teller distortions due to partial occupation of pi bonding orbitals formed from the metal d orbitals. Strong ferromagnetic couplings are predicted between nearest neighbor irons, as well as a small energy gap, yielding a good candidate for single molecule magnetism.

## Introduction

**Figure 1:**
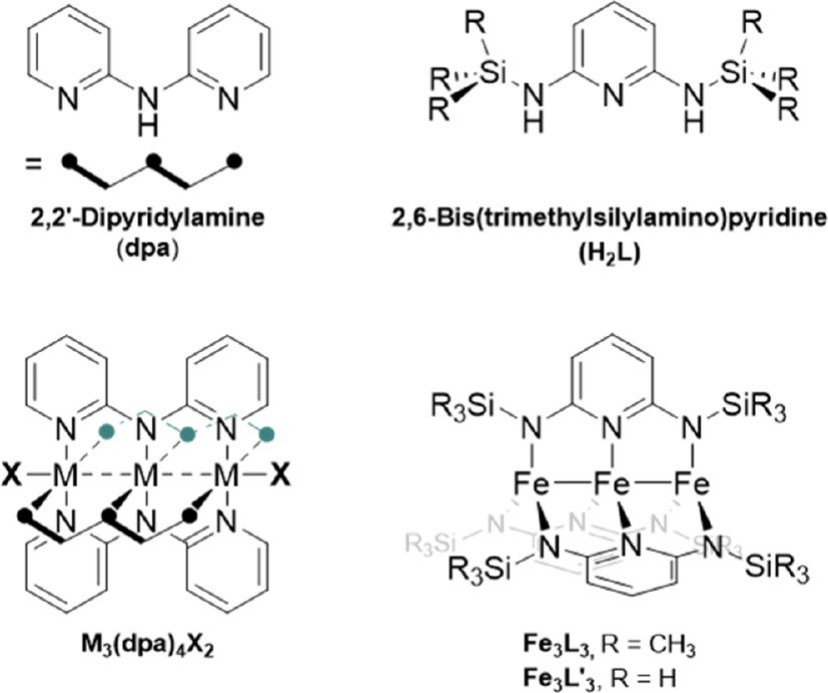
Structural motifs for the M_3_(dpa)_4_X_2_ (left) and **Fe**_**3**_**L**_**3**_ (right) EMACs. This study explores both the experimentally isolated molecule, **Fe**_**3**_**L**_**3**_, and a simpler analogue that replaces methyl substituents with hydrogen atoms, **Fe**_**3**_**L′**_**3**_, in order to reduce the overall size of the complex. Both compounds lack axial ligation compared to the dpa-based EMACs reported in the literature.

Extended metal atom chain (EMAC) complexes, multinuclear metal complexes with a linear arrangement of metals and at times relatively short M–M distances, have been the subject of significant interest since the discovery of the linear Ni_3_(dpa)_4_Cl_2_ in 1968, where dpa is the di(2-pyridyl)amido anion, and structurally characterized in 1991.^1−3^ Even though hundreds of tetragonal, axially ligated EMAC complexes with dpa, of the form M_3_(dpa)_4_X_2_ (Figure 1), have been structurally characterized, there is a distinct lack of high spin derivatives, likely the result of the strong ligand field. Notably, there is also a conspicuous absence of an iron containing EMAC supported by dpa.^4,5^

Interest in clusters of multiple iron atoms often stems from the possibility of isolating high-spin ground states that may behave as single-molecule magnets (SMM). These clusters often contain Fe atoms at short distances with overlapping metal valence orbitals, and significant axial magnetic anisotropy. This is especially true as diferryl, mixed valent (II/I), and diferrous systems are known with the iron atoms at bonding distances (Fe–Fe = 2.127–2.611 Å) that exhibit maximal spin ground states of *S* = 3 to *S* = 4.^6−10^ Though EMAC’s of iron are rare there are some examples. Two recent tetrairon examples^11,12^ are supported by three oligo-α-pyridyl ligands, Fe_4_(tpda)_3_X_2_ where tpda is *N*^2^,*N*^6^-bis(pyridine-2-yl)pyridine-2,6-diamine and X is Cl^–^ or Br^–^, and are composed of two Fe_2_ units with Fe–Fe = 2.97–2.99 Å. The Fe_2_ units have ferromagnetic (FM) coupling (*J* = +10.1 and +15.4 cm^–1^) and intermolecular antiferromagnetic coupling. A triiron species [Fe_3_(DpyF)_4_]^2+^, where DpyF^–^ is dipyridylformamide, first reported in 1998^13^ as the PF_6_^–^ salt and then reevaluated in 2021^14^ as the BF_4_^–^ salt, has shorter Fe–Fe contacts of 2.774 Å at 100 K. This complex has marginally stronger FM coupling with *J* = +14.5 cm^–1^ and an *S* = 6 ground state, likely a result of the shorter Fe–Fe distance. All three of these complexes show SMM behavior. Other notable, higher nuclearity Fe clusters also have high ground spin states and short Fe–Fe bond lengths including a recent triiron example housed in an Sn_18_ cage,^15^ and some have also exhibited SMM properties like tri and hexanuclear systems studied by Betley,^16−19^ and a recent unsymmetric, mixed valent pentairon supported by an organic nanocage studied by Cornia and Berry.^20^

Recently, the first triferrous EMAC with short Fe–Fe contacts was synthesized and reported. The iron–iron bond distance (Fe–Fe = 2.442 Å) is suggestive of a weak single bond, according to formal shortness ratios.^21^ One major difference for this compound was the use of the 2,6-bis-(trimethylsilylamido)pyridine ligand (L) that resulted in a trigonal coordination environment and the molecular formula **Fe**_**3**_**L**_**3**_, Figure 1. This is a rare example of such a coordination environment. Furthermore, the charge balance of the **Fe**_**3**_**L**_**3**_ complex does not require anionic axial ligands, which is another common feature of previously reported EMACs containing transition metals.^5,22^ These axial ligands have been shown to influence the symmetry of the metal–metal bond distances,^23−25^ but are absent in **Fe**_**3**_**L**_**3**_ and this may impact the energy ordering of the *d*-orbital manifold. Room temperature experimental evidence suggests a high-spin, *S* = 6, ground state configuration for the triiron complex, indicating ferromagnetic coupling between the metal atoms. This matches the predicted coupling for a hypothetical, four-coordinate triferrous EMAC complex^26^ and previously isolated Fe-based EMAC complexes^11,12^ between nearest-neighbor irons. Furthermore, instead of adopting the expected *D*_3_ symmetry seen in solution from ^1^H NMR, the reported crystal structure shows *C*_2_ symmetry suggesting a symmetry breaking as the compound is cooled and crystallized. In order to better understand why **Fe**_**3**_**L**_**3**_ is unique compared to other EMACs and the origin of the lower symmetry crystal structure, electronic structure calculations are reported herein from both density functional theory (DFT) and complete-active space self-consistent field (CASSCF) methodologies to elucidate the ground state configuration and its influence on the molecular properties.

**Figure 2:**
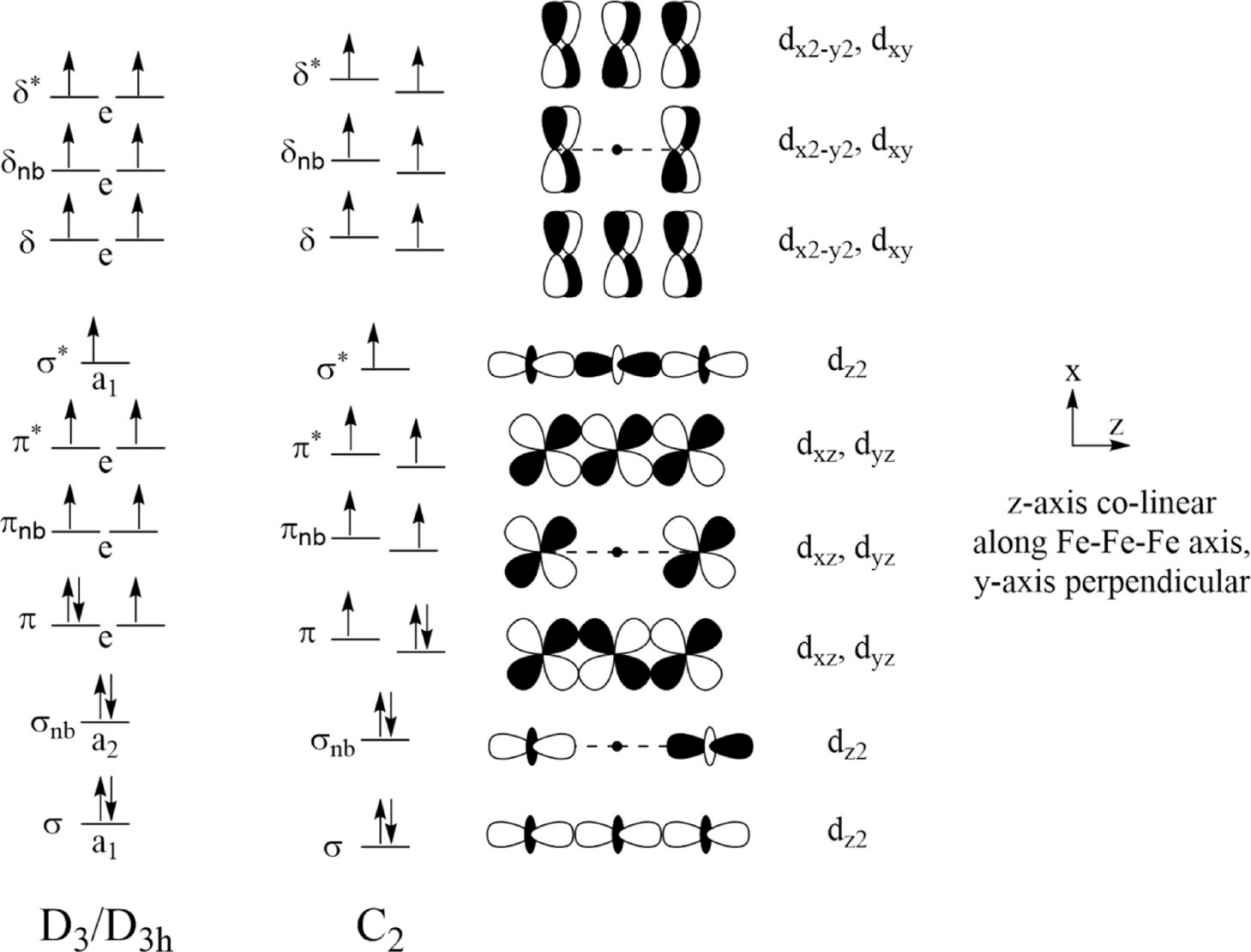
Molecular orbital diagram for the **Fe**_**3**_**L**_**3**_ and **Fe**_**3**_**L′**_**3**_ compounds in *D*_3(h)_ and *C*_2_ symmetry. Due to the lack of axial ligands, the sigma nonbonding orbital is lower in energy than the π orbital manifold. The (σ)^2^(σ_nb_)^2^(π)^3^(π_nb_)^2^(π*)^2^(σ*)^1^(δ)^2^(δ_nb_)^2^(δ*)^2^ configuration, referred to as the sigma nonbonding configuration throughout, is illustrated since this configuration will be shown to be the global ground state.

As will be demonstrated below, the root cause for this symmetry breaking lies in the occupation of molecular orbitals formed from linear combinations of the atomic iron *d* orbitals. Considering 3 metal centers with 5d orbitals and 6 electrons, it is possible to construct 15 molecular orbitals occupied with 18 electrons as shown in Figure 2. For the dpa-based EMACs, the sigma nonbonding orbital has been considered higher in energy than the orbitals of pi character.^5^ In **Fe**_**3**_**L**_**3**_, however, the lack of axial coordinating ligands will be shown to cause the sigma nonbonding orbital to drop below the pi manifold. The weak ligand field, a function of the L^2–^ donor strength and the low coordination number, likely plays a role in keeping the delta manifold low enough in energy to result in a high-spin configuration. Consequently we will be concerned with two electron configurations of **Fe**_**3**_**L**_**3**_. The first has the sigma nonbonding orbital fully occupied leading to partial occupation of the pi bonding orbitals: (σ)^2^(σ_nb_)^2^(π)^3^(π_nb_)^2^(π*)^2^(σ*)^1^(δ)^2^(δ_nb_)^2^(δ*)^2^. The second is an alternative configuration that leaves the sigma nonbonding orbital partially occupied while fully occupying the pi bonding orbitals: (σ)^2^(σ_nb_)^1^(π)^4^(π_nb_)^2^(π*)^2^(σ*)^1^(δ)^2^(δ_nb_)^2^(δ*)^2^. We will focus our discussion on the occupations of the σ, σ_nb_, and π orbitals, and will therefore refer to these two principal configurations using a truncated notation as the sigma nonbonding configuration, (σ)^2^(σ_nb_)^2^(π)^3^, and the pi bonding configuration, (σ)^2^(σ_nb_)^1^(π)^4^, with the implication that the other orbitals are occupied with the remaining α spin electrons shown in Figure 2.

Though multireference methods with perturbative treatment of correlation have yielded success in understanding the electronic structure and magnetic properties of EMACs in the literature,^24,25,27,28^ semilocal or hybrid DFT calculations are typically preferred for exploring the potential energy surface. The DFT results can then be compared with a higher level of theory for selected molecular configurations. Though commonly used functionals have been successful in transition metal chemistry,^6,−32^ they also have known challenges^28,33,34^ and higher-level DFT methods such as the random phase approximation (RPA) are becoming an alternative to wave function methods for cross-checking semilocal results. RPA is an attractive method for studying transition metal chemistry due to its elimination of self-interaction error in the exchange energy, nonempirical inclusion of dispersion interactions, and accurate treatment of small-gap systems due to its nonperturbative formulation.^35−40^ With efficient implementations making the computational cost of RPA close to that of semilocal functionals, RPA can be applied to large molecular systems and has previously been shown to yield accurate results for both energetics and geometries of transition metal and lanthanide systems.^35,41−48^ For a given spin state, RPA should yield qualitatively similar results as higher-level methods. The accuracy of RPA for predicting relative properties of different spin states in large complexes is still an open question and therefore best explored with multireference methods at this time.

Often the most important details of these EMAC complexes arise from the core of the molecule where the metals reside. In order to simplify the electronic structure calculations, a model complex, **Fe**_**3**_**L′**_**3**_—Figure 1, was created by swapping the methyl groups on the silyl methyl substituents (−SiMe_3_) for hydrogen atoms (−SiH_3_) in the pyridine ligand, which will be referred to as L′. This reduces the total number of atoms by nearly a factor of 2, and enables more careful control over the point group symmetry of the model complex. This small model ultimately exhibited analogous pertinent features of the full molecule, including following the same molecular orbital diagram shown in Figure 2. Therefore, many of the results of this study will focus on the smaller model, and tie them to the real complex whenever possible.

The organization of the paper is as follows. The computational details are first presented. Then DFT results for the full **Fe**_**3**_**L**_**3**_ complex are reported and compared to the available experimental data such as the molecular structure, Mössbauer isomer shifts, and ultraviolet/visible (UV/vis) spectra. The model complex, **Fe**_**3**_**L′**_**3**_, is then explored in *D*_3*h*_, *D*_3_, and *C*_2_ point group symmetries using semilocal, hybrid, and RPA density functional methods. After completing the analysis of these high-symmetry structures, a two-dimensional potential energy surface for different fixed iron–iron bond lengths is presented. Multireference calculations were also carried out on select structures to compare with the DFT results. Magnetic coupling constants are also reported for both **Fe**_**3**_**L**_**3**_ and **Fe**_**3**_**L′**_**3**_. All together, these different levels of theory synergize with one another to paint a clearer picture of the ground state properties of **Fe**_**3**_**L**_**3**_, and further illustrate the ability of RPA to act as a higher-level check on semilocal DFT results.

## Computational Details

Semilocal DFT and RPA calculations were carried out using a developer’s version of Turbomole v7.6,^49^ while multireference calculations were completed using ORCA v5.0,^50^ all in the gas phase. Using the published crystal structure as the initial guess, structural optimizations for **Fe**_**3**_**L**_**3**_ were performed using a combination of def2-TZVP basis sets for iron atoms with def2-SVP basis sets^51,52^ for all other atoms. The TPSS meta-GGA functional^53^ and the one-parameter hybrid, TPSSh,^54^ were used to relax the full structure without any symmetry constraints, though they converged to essentially *C*_2_ symmetric minima. Optimized structures were confirmed to be potential energy minima through an analytical force constant calculation.^55^ The *C*_1_ structures were then reoptimized using TZVP basis sets for all atoms and enforcing *C*_2_ symmetry in order to compare with the crystal structure. An additional all-electron, structural optimization using RPA@TPSSh was then performed. As explained in the Supporting Information, the specific combination of RPA with a semilocal functional will be referred to as RPA@DFT, and is needed to distinguish how the RPA energy is computed. All reported DFT results are for the *S* = 6, high-spin state (unless otherwise mentioned) and show little spin-contamination. Mössbauer isomer shifts^56^ and magnetic coupling constants^26^ were calculated using TPSSh with TZVP basis sets, while time-dependent DFT^57^ (TDDFT) was used to calculate the UV/vis spectra at this same level of theory. *J*-couplings were also calculated with RPA@TPSSh. High-symmetry calculations on the model **Fe**_**3**_**L′**_**3**_ were also performed using the same levels of theory, but with additional functionals (BP86,^58^ r^2^SCAN,^59^ B3LYP^60^) to ensure a consistent description across functionals. Resolution of the identity^61^ and the corresponding auxiliary basis sets^62,63^ were used to accelerate the computation of the two-electron Coulomb repulsion integrals in both the SCF, excited state, and correlation treatments. Two-dimensional potential energy surfaces for **Fe**_**3**_**L′**_**3**_ were calculated without symmetry constraints to explore the landscape of distortions for the triferrous core. Full details can be found in the Supporting Information.

Multireference calculations were carried out on **Fe**_**3**_**L′**_**3**_ utilizing the complete active-space self-consistent field (CASSCF) approach^64^ with a perturbative treatment of correlation provided by NEVPT2.^65^ Quasi-restricted orbitals were generated using the TPSS functional with Tight convergence thresholds and the RI-J approximation^66^ as input to the CASSCF calculations. The full *d*-orbital manifold of 18 electrons in 15 orbitals was selected for the active space of the three Fe^2+^ ions. State-averaged CASSCF calculations were then performed using 29 states from the *S* = 6 manifold, followed by an additional calculation including 28 states with *S* = 5. The RI-JK approximation and basis sets^63,67,68^ were applied to accelerate the calculation of two-electron integrals in both the SCF and correlation treatment.

## Results

### **Fe**_**3**_**L**_**3**_ Results

**Figure 3:**
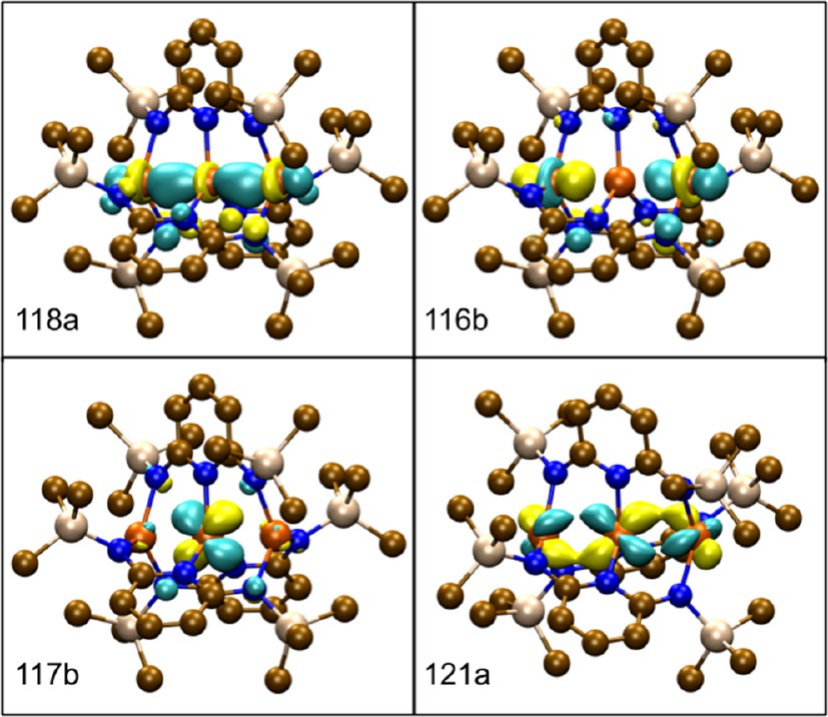
Frontier β-spin molecular orbitals for **Fe**_**3**_**L**_**3**_ calculated using TPSSh with def2-TZVP basis sets. The HOMO β-spin orbital does show pi-character (117b), but the sigma nonbonding orbital (116b) is lower in energy and also occupied along with the sigma bonding orbital (118a). The second pi bonding orbital (121a) corresponds to the LUMO; the structure was rotated to best show the orbital. A contour value of 0.05 was used for each plot and the hydrogen atoms have been suppressed for clarity.

The initial geometry optimizations utilizing TPSS and TPSSh with def2-SVP/TZVP basis sets converged to minima with quasi-*C*_2_ symmetry, even though symmetry was not enforced in the calculation. The crystal structure itself also exhibited *C*_2_ symmetry, so the initial calculation was consistent with the experimental results. It was hypothesized that in *D*_3_ symmetry the ground state would correspond to the pi bonding configuration, (σ)^2^(σ_nb_)^1^(π)^4^, defined in the Introduction.^21^ This concept is consistent with the general orbital orderings found in the dpa-type EMAC compounds reported in the literature;^5,24^ however, it is not consistent with the frontier molecular orbitals obtained from the TPSS(h) calculations, Figure 3.

As seen in Figure 3, the frontier β spin MOs reflect the sigma nonbonding configuration defined in the Introduction, (σ)^2^(σ_nb_)^2^(π)^3^, with the remaining electrons occupying the other σ, π, and δ orbitals as shown in Figure 2. Instead of fully occupying two pi bonding orbitals, leaving the sigma nonbonding orbital partially occupied, the sigma nonbonding orbital is lower in energy and therefore populated first by the β spin electrons. This results in only 1 β spin electron occupying the pi bonding orbitals, compared to the hypothesized ground state suggested in ref (21), leading to a B-type state due to the symmetry of the HOMO β-spin orbital. In the absence of axial ligands, the energy of the sigma nonbonding orbital drops below the pi bonding orbitals. The occupation of the sigma nonbonding orbital is likely one of the reasons why the Fe–Fe bond lengths are as long as they are, compared to shorter bond lengths expected from a stronger metal–metal bonding through occupation of only the sigma and pi bonding orbitals.

The optimized geometries from TPSS and TPSSh with TZVP basis sets are in excellent agreement with the crystal structure, lending further credibility to the frontier MOs. The reported Fe–Fe bond lengths in the crystal are 2.442 Å, while TPSS yields 2.415 Å and TPSSh yields 2.456 Å, Table 1. The Fe–N distances were also comparable, with TPSS & TPSSh producing distances between 1.98 and 2.00 Å, compared to the average 1.985 Å distance reported for the crystal. Other configurations with lower spin were calculated using broken-symmetry DFT,^69^ but these states were all higher in energy than the *S* = 6 state, indicating the *S* = 6 state is the ground state for this complex. This prediction is consistent with the Evans method experiments that indicated a high-spin, ferromagnetically coupled magnetic susceptibility at 300 K. The coupling of the metal centers is provided primarily by the sigma bonding orbitals, which are delocalized over the triiron core. This torsion of the ligands along the Fe_3_–Fe_1_–Fe_2_ axis reduces the overlap that the metal d orbitals have with one another and could be another contributing factor to the weak metal–metal bonding. The dihedral angle of the pyridine ligands in the optimized structures, measured as N–Fe_3_–Fe_2_–N for a given ligand, is approximately 45° which is a significant deviation from an ideal *D*_3*h*_ geometry.

**Table 1:** Optimized Structural Information for **Fe**_**3**_**L**_**3**_ Obtained from Semilocal and RPA Calculations Using TZVP Basis Sets Compared to the Experimental Crystal Structure^a^

**Fe**_**3**_**L**_**3**_	Fe–Fe distance (Å)	Avg Fe–N distance (Å)	Avg pyridine dihedral angle (deg)
TPSS	2.415	1.994	45.7
TPSSh	2.456	1.995	44.7
RPA@TPSSh	2.473	1.984	47.0
Crystal	2.442	1.985	45.5

a The DFT and RPA optimized structures are in good agreement with the reported metrics from the crystal structures. The average RPA Fe–Fe bond length is reported to simplify the comparison.

After completing the geometry optimization with TPSSh, another optimization was started using RPA@TPSSh with def2-TZVP basis sets for all atoms. If the resulting structure is very similar to the semilocal results then it indicates that dispersion and strong correlation effects are not playing an outsized role in determining the optimized structure. Fortunately this is the case and RPA@TPSSh yields Fe–Fe distances of 2.471 and 2.475 Å, and Fe–N distances between 1.97 and 2.00 Å, which is very similar to the crystal structure and semilocal functional results. The Fe–Fe distances are not perfectly symmetrical in this case, owing to the lack of symmetry enforced in the calculation, but are less than 0.5 pm different. Lastly, the dihedral angles of the pyridine ligands range between 42 and 50°, which also agree with the angles seen in the crystal structure. RPA is known to yield bond lengths that tend to be slightly longer than those obtained with semilocal functionals, so the results for the **Fe**_**3**_**L**_**3**_ compound are in line with previous calculations on other transition metal and lanthanide compounds.^35,41−43^ Furthermore, the agreement between RPA and semilocal results confirms the semilocal description of **Fe**_**3**_**L**_**3**_ and the predicted high-spin, sigma nonbonding ground state occupation, (σ)^2^(σ_nb_)^2^(π)^3^.

To go beyond a comparison of the molecular geometries for **Fe**_**3**_**L**_**3**_, Mössbauer isomer shifts and UV/vis spectra were also computed for comparison with the available experimental data. Utilizing the calibration constants in ref (56), TPSSh predicts the isomer shifts for Fe_1_ and Fe_2,3_ to be 0.52 and 0.44 mm s^–1^, respectively, at the *C*_2_ geometry optimized with TZVP basis sets. These values are in excellent agreement with the 80 K solid sample measurements reported in ref (21), which are 0.52 and 0.46 mm s^–1^ for Fe_1_ and Fe_2,3_, respectively. These same isomer shifts were also evaluated on a representative *D*_3_ symmetric structure for **Fe**_**3**_**L**_**3**_, which yielded an isomer shift of 0.44 mm s^–1^ and 0.40 mm s^–1^ for Fe_1_ and Fe_2,3_, see Supporting Information for more details. While the shifts for Fe_2,3_ on the ends of the linear chain are not as sensitive to the change in symmetry, Fe_1_ (the central ion) shows a change of 0.08 mm s^–1^ in the predicted isomer shift. As will be explained below, this reduction in symmetry as the temperature is lowered causes the molecule to transition from *D*_3_ to *C*_2_ symmetry due to Jahn–Teller distortions. The agreement between the Mössbauer isomer shifts is another reflection of the sigma nonbonding configuration adopted by **Fe**_**3**_**L**_**3**_, as the pi bonding configuration likely yields smaller isomer shifts (Supporting Information), and further supports the MO diagram in Figure 2.

**Figure 4:**
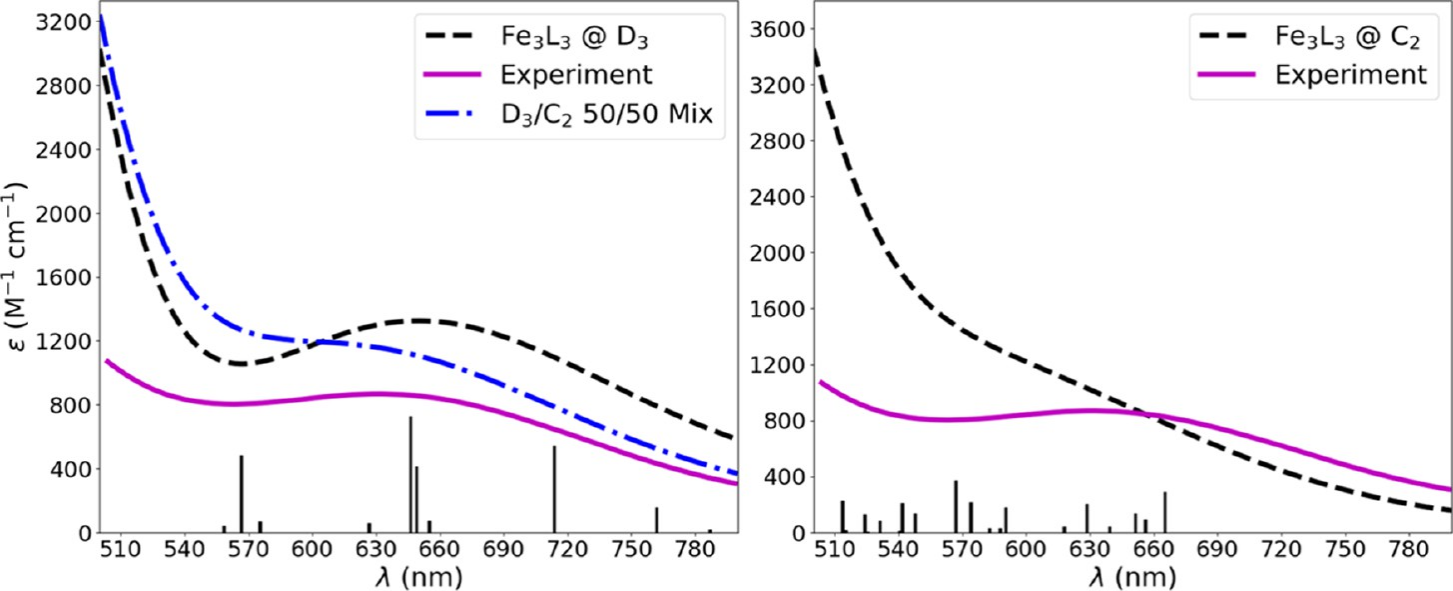
Simulated gas phase UV/vis spectra from TPSSh with SVP/TZVP basis sets for **Fe**_**3**_**L**_**3**_ in *D*_3_ (left) and *C*_2_ (right) point groups overlaid with the experimental spectrum obtained in THF. While the *D*_3_ structure does exhibit a shape that more closely resembles the experimental spectrum, the *C*_2_ spectrum cannot be ruled out due to the accuracy limits of TDDFT. A simple 50/50 mixture of the two predicted spectra is also shown (left). The heights of the discrete excitations (indicated as vertical bars) have been scaled by 1.5 to improve visibility, but no changes were made to the predicted extinction coefficient.

The gas phase, zero temperature UV/vis spectra were simulated using TDDFT excitation energy calculations at the *C*_2_ and *D*_3_ optimized structures for **Fe**_**3**_**L**_**3**_ obtained with TPSSh, Figure 4. Analysis of the excitations shows a mixture of excitations originating from the ligand amine nitrogens and the metal d orbitals terminating in unoccupied *d*orbitals between 400 and 800 nm, see the Supporting Information for more information. The spectra between 350 and 500 nm are very similar for *D*_3_ and *C*_2_ symmetries, so we have focused our analysis on the 500 to 800 nm range of the UV/vis spectra. Comparing the theoretical spectra to the experimental spectra obtained at a concentration of 3.5 × 10^–5^ M, the *C*_2_ spectra does not have a pronounced maximum between 600 and 700 nm while the *D*_3_ spectra shows a clearly defined maximum in this region. The *D*_3_ spectra appears to be a better match due to the presence of excitations above 690 nm and the progression of intensities from long to short wavelengths. However, given the accuracy limits of TDDFT for peak positions and intensities,^70^ it is not possible to say conclusively that one point group is correct over the other in comparison to the experimental UV/vis spectrum at room temperature since both states will be sampled during molecular vibrations. In fact, a simple 50/50 mixture of the two predicted spectra also resembles the experimental spectrum reasonably well.

Even though these spectra do not provide conclusive evidence for one structure over the other at room temperature, it still serves to confirm the assignment of the ground state to the sigma nonbonding configuration since both spectra are a reasonable match to the experiment. To get more insight into the preference for one structure over another, it is pertinent to use a model where more precise control of the geometry is possible. We therefore turn to the model system, **Fe**_**3**_**L′**_**3**_, where it is straightforward to have tight control over the molecular symmetry.

### **Fe**_**3**_**L′**_**3**_ Results

#### High-Symmetry Exploration with Semilocal Functionals

**Figure 5:**
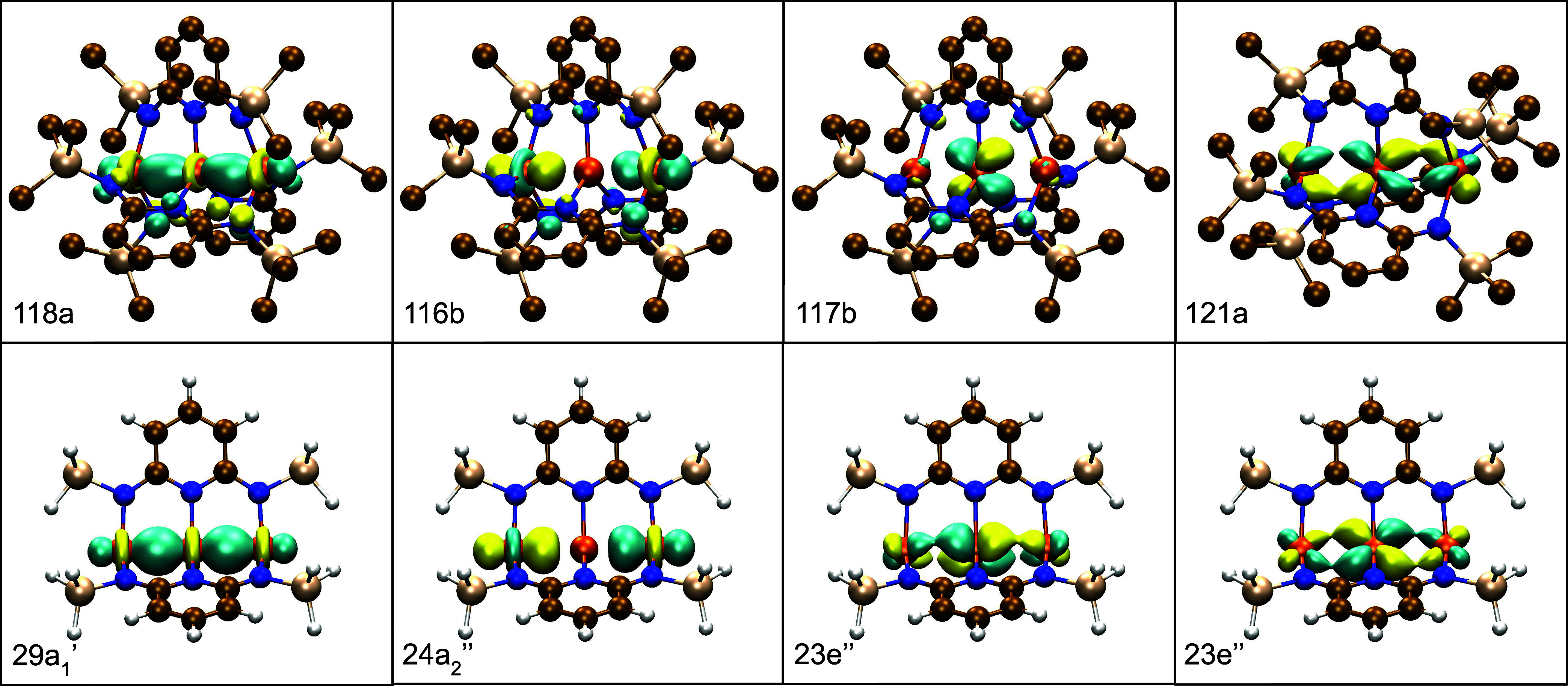
Frontier β-spin molecular orbitals for **Fe**_**3**_**L′**_**3**_ (bottom) calculated using TPSS in *D*_3*h*_ symmetry. The overall character of these orbitals matches the previous MOs obtained for the full **Fe**_**3**_**L**_**3**_ complex (top). The pi bonding orbitals (23e″) are fractionally occupied in this symmetry group due to their degeneracy and correspond to the HOMO. The sigma-type orbitals are both fully occupied and lower in energy than the pi bonding orbitals. A contour value of 0.05 was used for each plot.

The **Fe**_**3**_**L′**_**3**_ model was first constructed in *D*_3*h*_ symmetry and then optimized with each functional. The optimized geometries for the model complex using TZVP basis sets for all atoms are summarized in Table 2. Comparing the TPSS and TPSSh results between the model and real complex illustrates the applicability of **Fe**_**3**_**L′**_**3**_ for describing the triferrous core of the molecule, as the Fe–Fe and Fe–N distances resemble those from **Fe**_**3**_**L**_**3**_ though the Fe–Fe distances have increased by a few pm. The frontier MOs, Figure 5, also matched what had been seen in the **Fe**_**3**_**L**_**3**_ complex; the sigma nonbonding orbital is occupied and lower in energy than the pi bonding orbitals, leaving only one β-spin electron to occupy the degenerate pi bonding orbitals, see Figure 2. The partial occupation and degeneracy of the pi bonding orbitals gives rise to an E-type state, which is susceptible to a Jahn–Teller distortion in order to lift the degeneracy. After computing the vibrational modes, a saddle point was obtained with imaginary modes that tend to lower the symmetry of the compound by introducing torsion along the ligands, therefore lowering the symmetry from *D*_3*h*_ to *D*_3_.

**Table 2:** Fe–Fe Distances and Average Fe–N Distances (Å), as well as Average Pyridine Dihedral Angles (deg), Obtained for **Fe**_**3**_**L′**_**3**_ in *D*_3h_, *D*_3_, and *C*_2_ Point Groups with the Tested Functionals Using TZVP Basis Sets for All Atoms^a^

	*D*_3*h*_			*D*_3_			*C*_2_		
**Fe**_**3**_**L′**_**3**_	*d*(Fe–Fe) (Å)	*d*(Fe–N) (Å)	pyridine dihedral	*d*(Fe–Fe) (Å)	*d*(Fe–N) (Å)	pyridine dihedral	*d*(Fe–Fe) (Å)	*d*(Fe–N) (Å)	pyridine dihedral
BP	2.470	1.988	0.0	2.451	1.986	25.2	2.455	1.986	25.4
TPSS	2.463	1.988	0.0	2.451	1.985	24.5	2.463	1.986	24.8
r^2^SCAN	2.453	1.982	0.0	2.439	1.980	23.1	2.451	1.980	23.4
TPSSh	2.484	1.990	0.0	2.467	1.987	25.5	2.503	1.988	25.3
B3LYP	2.491	1.992	0.0	2.482	2.005	26.8	2.526	2.006	26.4
**Fe**_**3**_**L**_**3**_ xtal^21^							2.442	1.985	45.5

a The experimental results for **Fe**_**3**_**L**_**3**_ are reported for comparison, and the similarity between the model and full complex is apparent.

**Figure 6:**
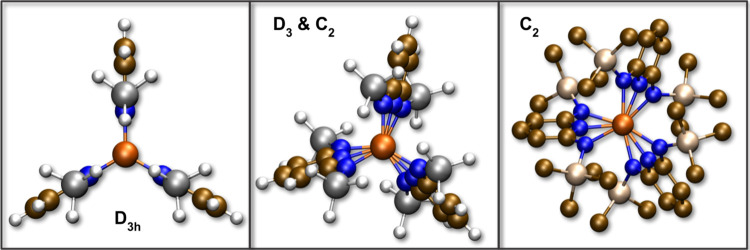
Axial view of **Fe**_**3**_**L′**_**3**_ illustrating the development of the pyridine dihedral angle along the Fe_3_–Fe_1_–Fe_2_ axis in *D*_3_ and *C*_2_ symmetry (middle) compared to the ideal *D*_3*h*_ geometry (left). This dihedral angle is ∼25° for **Fe**_**3**_**L′**_**3**_ and increases to ∼45° in **Fe**_**3**_**L**_**3**_ (right) with the bulkier ligands. Hydrogen atoms have been suppressed for clarity for **Fe**_**3**_**L**_**3**_.

The resulting *D*_3_ structures were reoptimized using the same level of theory. The frontier MOs are completely consistent with their *D*_3*h*_ counterparts. The Fe–Fe and Fe–N distances change marginally, however a torsion angle develops along the ligands, Table 2. The dihedral angle along the pyridine ligands is ∼25°, Figure 6, which is smaller than the ∼45° angle found in the real complex, but is still a significant twist compared to *D*_3*h*_. The difference in dihedral angles is certainly due to the bulkiness of the methyl substituents in **Fe**_**3**_**L**_**3**_ compared to the hydrogens in **Fe**_**3**_**L′**_**3**_, and does not significantly impact the Fe–Fe distances or frontier MOs. To test this hypothesis, successive methyl groups were added to the **Fe**_**3**_**L′**_**3**_ model compound in order to track the changes in the dihedral angle. For one methyl group, the dihedral angle remains close to the proteo form (∼25°), while for two methyl groups the dihedral angle increases to ∼30°. By adding the third methyl group, the complex contorts further to attain the ∼45° angle seen in the crystal structure. Please see the Supporting Information for further details and Figure S1 for a space-filled model of **Fe**_**3**_**L**_**3**_ in *D*_3*h*_ and *D*_3_ symmetry.

Comparing the energies of the *D*_3*h*_ and *D*_3_ structures, lowering the symmetry stabilized the compound by ∼2 kcal/mol for all utilized semilocal functionals. Since the pi bonding orbitals are still degenerate and partially occupied, further relaxation to lower symmetry is needed to lift this degeneracy. In fact some functionals predict the optimized *D*_3_ structures to have small imaginary vibrational modes that reduce the symmetry further to *C*_2_. This further lowering of symmetry is due to a Jahn–Teller effect that breaks the degeneracy of the pi bonding orbitals due to the trigonal distortions of a few degrees around the metal ions.

**Figure 7:**
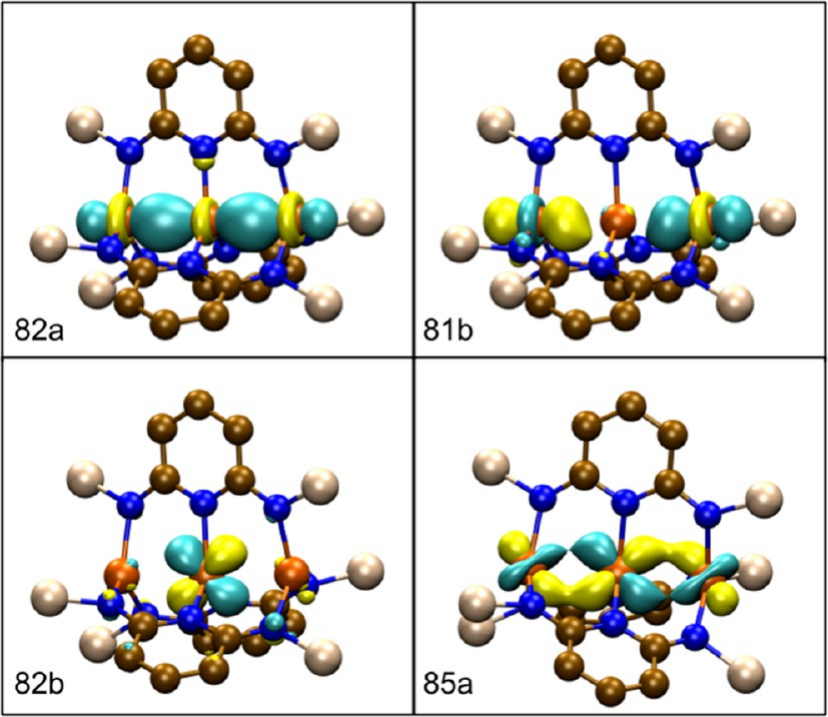
Frontier β-spin molecular orbitals for **Fe**_**3**_**L′**_**3**_ calculated using TPSSh in *C*_2_ symmetry. The overall character of these orbitals matches the previous MOs obtained for the full **Fe**_**3**_**L**_**3**_ complex in Figure 3. The 82b orbital corresponds to the HOMO while 85a corresponds to the LUMO. A contour value of 0.05 was used for each plot and the hydrogen atoms have been suppressed for clarity.

In *C*_2_ symmetry the degeneracy of the pi bonding orbitals is lifted therefore eliminating the fractional occupation encountered in higher symmetry groups. The optimized molecular structures in *C*_2_ symmetry are very close to the ones obtained in *D*_3_ symmetry; however, the *C*_2_ structures were all confirmed to be potential energy minima due to the elimination of imaginary vibrational modes. These *C*_2_ symmetric structures contain distortions away from trigonal symmetry in the N–Fe–N angles around each metal center by approximately 1–3° and increased Fe–Fe bond lengths by 2 to 5 pm compared to *D*_3_. Energetically the *D*_3_ and *C*_2_ structures are within 0.5 kcal/mol of one another if both structures are calculated in *C*_2_ symmetry to avoid the fractional occupation of the pi orbitals. Taken together, these results imply that the Jahn–Teller effect does not lead to a significant geometric shift or energetic stabilization. The pertinent molecular orbitals are plotted in Figure 7 and they closely resemble the orbitals for **Fe**_**3**_**L**_**3**_.

Mössbauer isomer shifts were also calculated for the **Fe**_**3**_**L′**_**3**_ system in *D*_3_ and *C*_2_ symmetry with TZVP basis sets. Just as for **Fe**_**3**_**L**_**3**_, the isomer shifts predicted in *C*_2_ symmetry are a much better match to the experimental data than those from the *D*_3_ structure. For Fe_1_ and Fe_2,3_ the predicted isomer shifts are 0.57 and 0.42 mm s^–1^ in *C*_2_ symmetry, respectively, while these same shifts are predicted to be 0.51 and 0.40 mm s^–1^ in *D*_3_ symmetry. This reduction in the isomer shifts was also seen for the **Fe**_**3**_**L**_**3**_ compound described above, and is further indication of the applicability of the model complex and the consistency of the semilocal DFT results. To go beyond the semilocal level, RPA geometry optimizations were also performed, however these calculations led to some interesting complications and subsequent results.

Before discussing the RPA results, we note that an alternative *D*_3_ electron configuration was tested by promoting an electron from the 24a_2_″ (sigma nonbonding) orbital to the 23e″ orbitals (pi bonding), Figure 5, leading to the pi bonding configuration, (σ)^2^(σ_nb_)^1^(π)^4^, originally hypothesized for **Fe**_**3**_**L**_**3**_.^21^ The resulting calculations converged to an Aufbau configuration, except for the BP functional, however the resulting total energy of this state was higher compared to the *D*_3_ structure obtained with the sigma nonbonding configuration, (σ)^2^(σ_nb_)^2^(π)^3^. Furthermore, the Fe–Fe bond lengths universally contracted compared to the previously optimized structures, as expected from the increased bond order, leading to bond lengths that were at least 0.1 Å shorter than those obtained with the sigma nonbonding configuration. Mössbauer isomer shifts predicted for **Fe**_**3**_**L′**_**3**_ with the alternative pi bonding configuration yield much larger discrepancies compared to the experimental data as well. This is further indication that the pi bonding configuration, (σ)^2^(σ_nb_)^1^(π)^4^, is unlikely, as it would lead to significantly shorter Fe–Fe distances than obtained in the experimental crystal structure and smaller Mössbauer isomer shifts. Results for this alternative occupation are reported in the Supporting Information, but will not be considered further here.

#### Structural Optimization and Energetic Comparisons from RPA

In order to more thoroughly verify the semilocal DFT results for the **Fe**_**3**_**L′**_**3**_ compound, RPA calculations were also performed utilizing different semilocal functionals to generate the reference state. RPA geometry optimizations were carried out in *C*_1_ symmetry starting from the *C*_2_ optimized structures for each reference functional using SVP/TZVP basis sets. Unfortunately, the optimized Fe–Fe distances reported in Table 3 show some variation dependent upon the reference state. For pure semilocal functionals such as BP and TPSS, the optimized Fe–Fe distances are asymmetric in the chain, leading to Fe–Fe bond lengths that differ by ∼0.13 Å. For hybrid functionals such as TPSSh and B3LYP, the optimized RPA geometry has symmetric Fe–Fe distances of ∼2.495 Å, retains the *C*_2_ symmetry of the molecule, and is in very good agreement with the experiment. At face value this seems to imply that RPA is not an accurate method here since the functional dependence produces different results for the minimum of the PES. However, asymmetric structures have been found in other EMAC complexes that were shown to have a relatively flat potential energy surface close to the minimum.^24,27,71,72^ This could lead to a situation where the asymmetric and symmetric structures are quite close in energy and the preference for one or the other would be influenced by the reference functional used in the RPA calculation. In order to explore alternative geometries, 2D potential energy surfaces can be calculated using a semilocal functional and then the RPA calculations added on top. A similar idea was used for DFT + CASSCF in a previous study of EMACs containing chromium.^24^

**Table 3:** RPA Optimized Structures for **Fe**_**3**_**L′**_**3**_ Using Different Reference Functionals with SVP/TZVP Basis Sets in *C*_1_^a^

**Fe**_**3**_**L′**_**3**_	Fe–Fe distances (Å)	Avg Fe–N distance (Å)	avg pyridine dihedral angle (deg)
RPA@BP	2.414	1.996	32.5
	2.547		
RPA@TPSS	2.405	1.993	32.2
	2.544		
RPA@TPSSh	2.495	1.994	31.2
RPA@B3LYP	2.509	1.995	31.0

a The reference functional dependence leads to different predictions for the optimized structure, with hybrid functionals maintaining the quasi-*C*_2_ symmetry of the reference functional, while RPA evaluated with semilocal functionals indicates an asymmetric potential energy minimum with differences in Fe–Fe bond lengths of ∼0.14 Å.

#### 2D PES from DFT and RPA

To get a better picture of the energetics of distortion for the triferrous core, 2D potential energy surfaces were calculated as described in the Supporting Information. A reasonable range of bond lengths were chosen to encompass the minima predicted by different functionals and to sample stretched and compressed configurations as well. Symmetry was not enforced during these calculations. The geometries for each functional, optimized with TZVP basis sets at each point on the PES, are available in the electronic Supporting Information in XYZ format.

**Figure 8:**
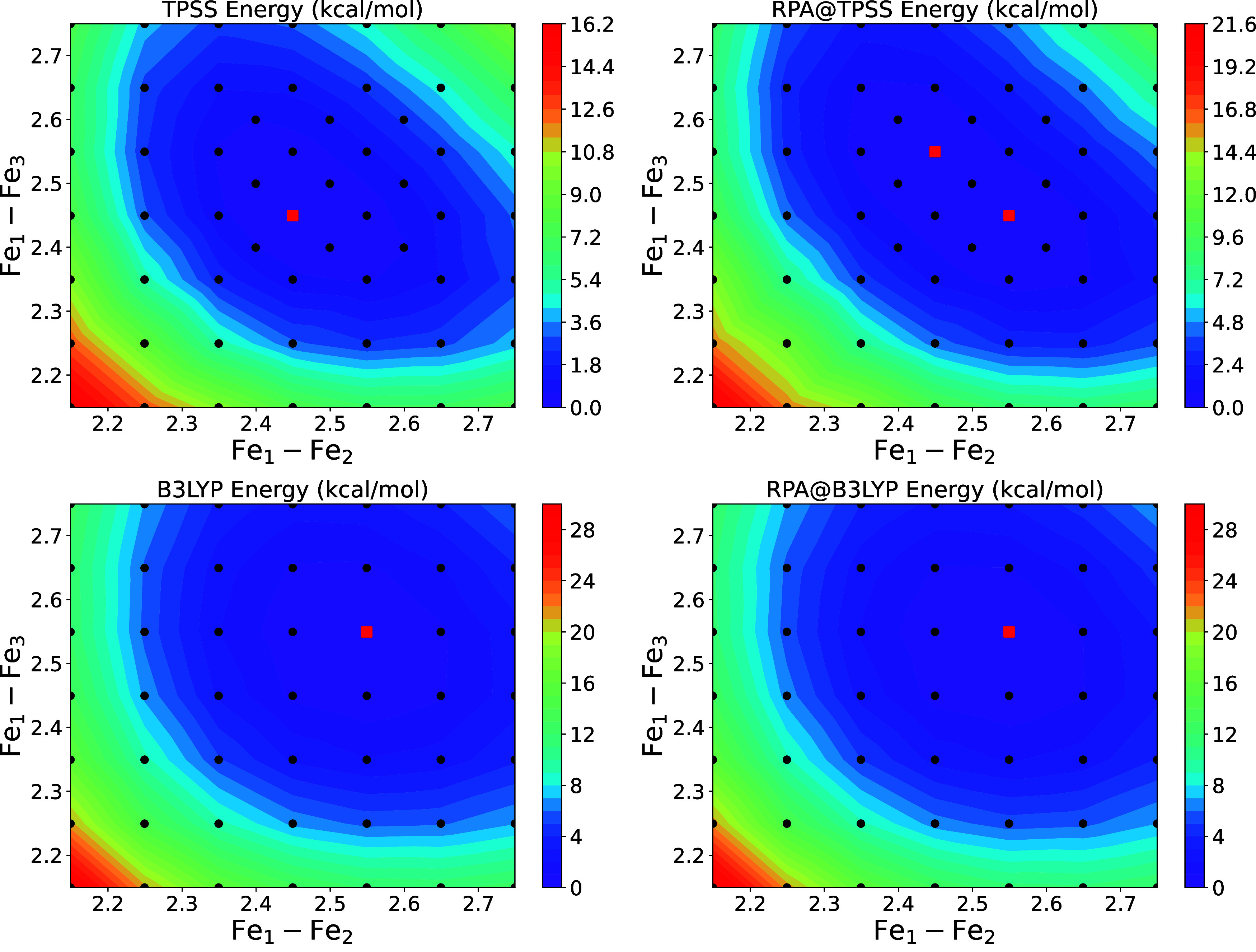
2D PES calculated for TPSS, B3LYP, and RPA using def2-TZVP basis sets. The PES is relatively flat near the minimum, with symmetric and asymmetric configurations being within less than 1 kcal/mol of one another. The minimum of each surface is indicated by a red square.

Contour plots of the results for TPSS, and B3LYP, as well as the RPA results evaluated with each reference determinant, are reported in Figure 8. The minimum energy point is indicated by a red square. As seen in Tables 2 and 3, symmetric minima are obtained for the DFT and hybrid functionals, and the minimum energy predicted by RPA depends on the reference functional. Inspecting the PES, it is clear there are a range of Fe–Fe bond lengths, both symmetric and asymmetric, that are within less than 1 kcal/mol of one another and therefore the triferrous core can be expected to fluctuate between different structures at finite temperature in solution as seen for previous EMACs.^72^ This small energy difference approaches the accuracy limits of even RPA, however given the general consistency between calculations for the PES with the different methods, the RPA results overall confirm the trends observed with semilocal functionals. While the DFT and RPA results are consistent with one another at multiple levels of theory for describing the molecular geometry, and indicate an *S* = 6 ground state, it is still pertinent to explore energy differences between spin-states utilizing multireference methods.

#### Multireference Results

In order to investigate the electronic structure of multiple spin states, CASSCF with perturbative correlation from NEVPT2 was applied to select molecular configurations. These calculations were carried out on both the *D*_3_ and *C*_2_ structures obtained with BP using SVP/TZVP basis sets. The same basis sets were utilized for the CASSCF calculations as were used for the DFT calculations.

The *D*_3_ structure is orbitally degenerate due to the degeneracy of the pi bonding orbitals and this is immediately seen in the CAS calculations. Performing state-averaged calculations containing any number of states for the *S* = 6 manifold yielded two degenerate states as the ground state, each with double occupation of 1 pi bonding orbital. Including the *S* = 5 manifold breaks this degeneracy slightly, increasing the energy difference between states to 27 cm^–1^, and changing the dominant configurations that comprise the ground state. Including NEVPT2 marginally decreases this difference to 15 cm^–1^, and places the lowest energy *S* = 5 state 190 cm^–1^ above the *S* = 6 ground state. Looking at the *C*_2_ structure, the state-averaged CASSCF calculations containing *S* = 6 and *S* = 5 states indicate that the lowest energy configuration still corresponds to the *S* = 6 manifold, but there are excited states of both spin characters within 300 cm^–1^ of the ground state. For instance, using just *S* = 6 with 29 states in the calculation, the first excited state is 295 and 203 cm^–1^ higher than the ground state with and without NEVPT2. Incorporating the *S* = 5 states into the calculation, the lowest energy state of this manifold drops below the first excited state of *S* = 6 manifold. The energy difference between the lowest energy state of *S* = 6 and the lowest energy state of *S* = 5 is 193 and 110 cm^–1^ with and without NEVPT2 corrections. While these calculations indicate that different spin-state manifolds are very close in energy, which could influence the SMM properties of **Fe**_**3**_**L**_**3**_, they also confirm the semilocal and RPA calculations that predicted the high-spin state should be the global ground state of the **Fe**_**3**_**L′**_**3**_ model and the full **Fe**_**3**_**L**_**3**_ compound.

Looking at the converged active space orbitals reveals strong similarities to the DFT orbitals shown in Figures 3, 5, and 7. Plots of all the active space orbitals can be found in the Supporting Information, Figure S2. Since the difference in *D*_3_ and *C*_2_ structures was minimal, as well as the differences in the orbitals, only the plots for the *C*_2_ structure are included. Inspecting the configuration weights, a single configuration corresponding to the sigma nonbonding configuration, (σ)^2^(σ_nb_)^2^(π)^3^(π_nb_)^2^(π*)^2^(σ*)^1^(δ)^2^(δ_nb_)^2^(δ*)^2^, is the dominant contribution for the ground state, Table 4, with either state averaged calculation. Two other configurations are noteworthy, and each occupies antibonding pi or sigma orbitals opposed to additional bonding orbitals. This is another piece of evidence to understand why the Fe–Fe bond lengths in this compound are as long as they are, as occupation of the antibonding orbitals would certainly reduce the bonding interaction between the irons.

**Table 4:** Dominant Configuration Weights (%) for **Fe**_**3**_**L′**_**3**_ in *C*_2_ Symmetry Calculated from State-Averaged CASSCF Calculations^a^

	*S* = 6	*S* = 6 and *S* = 5
configurations	29 states	29 and 28 states
(σ)^2^(σ_nb_)^2^(π)^3^(π_nb_)^2^(π*)^2^(σ*)^1^(δ)^2^(δ_nb_)^2^(δ*)^2^	55.4	77.4
(σ)^2^(σ_nb_)^2^(π)^2^(π_nb_)^2^(π*)^3^(σ*)^1^(δ)^2^(δ_nb_)^2^(δ*)^2^	23.0	<1
(σ)^1^(σ_nb_)^2^(π)^2^(π_nb_)^2^(π*)^3^(σ*)^2^(δ)^2^(δ_nb_)^2^(δ*)^2^	14.4	7.1

a The difference in *D*_3_ and *C*_2_ is minimal for the dominant configurations and support double occupancy of the sigma nonbonding orbital, and indicate some occupation of the antibonding orbitals.

#### Predicted *J*-Couplings

Given the close proximity of the *S* = 6 and *S* = 5 manifolds from the CASSCF calculations, magnetic coupling constants, *J*_12_ & *J*_23_, were calculated with DFT using a simple Ising model for multiple metal centers,^26,73,74^ details reported in the Supporting Information. Both TPSSh and RPA@TPSSh predict strong ferromagnetic coupling between the central Fe_1_ and the outer Fe_2(3)_ ions, and modest antiferromagnetic coupling between Fe_2_ and Fe_3_ for both **Fe**_**3**_**L**_**3**_ and **Fe**_**3**_**L′**_**3**_, Table 5. The ferromagnetic couplings are an order of magnitude larger than the hypothetical triferrous EMAC with axial ligation studied in ref (26). The strong ferromagnetic coupling constants further support the *S* = 6 ground state assignment, and indicate that the magnetic moment of the compound should be larger than 3 independent, *S* = 2 ferrous ions. Indeed this is the case, as the experimental magnetic moment for **Fe**_**3**_**L**_**3**_ was observed to be χ_M_*T* ∼ 20 emu K mol^–1^ at 300 K.^21^ Further experimental analysis of the variable temperature magnetic properties of **Fe**_**3**_**L**_**3**_ is certainly warranted based on our findings, since there are several indications this molecule illustrates SMM behavior.

**Table 5:** Predicted Magnetic Coupling Constants from a Simple Ising Model for Spin Centers Using TPSSh and RPA@TPSSh with TZVP Basis Sets^a^

	*J*_12_ and *J*_13_ (cm^–1^)		*J*_23_ (cm^–1^)	
	**Fe**_**3**_**L**_**3**_	**Fe**_**3**_**L′**_**3**_	**Fe**_**3**_**L**_**3**_	**Fe**_**3**_**L′**_**3**_
TPSSh	131	154	–53	–31
RPA@TPSSh	225	219	–24	–27

a The irons are arranged as Fe_3_–Fe_1_–Fe_2_ in the linear chain. The coupling constant *J*_12_ is equivalent by symmetry to *J*_13_. Strong ferromagnetic coupling is indicated for nearest-neighbors, while anti-ferromagnetic coupling is predicted for Fe_2_ and Fe_3_.

## Conclusions

Looking at the multireference results compared to the DFT results, all levels of theory paint a consistent picture of the electronic structure realized in **Fe**_**3**_**L**_**3**_. While many previous EMAC complexes of first-row transition metals have involved axial ligation, the absence of the axial ligands in **Fe**_**3**_**L**_**3**_ causes the sigma nonbonding orbital to drop below the pi bonding orbitals. This change in orbital ordering leads to a high-spin, orbitally degenerate ground state in *D*_3h_ & *D*_3_ symmetry that becomes nondegenerate in *C*_2_ after breaking the local trigonal symmetry around each Fe. Longer Fe–Fe distances are therefore the consequence of the reduced bond order in the sigma nonbonding configuration, (σ)^2^(σ_nb_)^2^(π)^3^(π_nb_)^2^(π*)^2^(σ*)^1^(δ)^2^(δ_nb_)^2^(δ*)^2^, compared to the originally hypothesized pi bonding configuration, (σ)^2^(σ_nb_)^1^(π)^4^(π_nb_)^2^(π*)^2^(σ*)^1^(δ)^2^(δ_nb_)^2^(δ*)^2^. Based on the results of the **Fe**_**3**_**L′**_**3**_ model, the symmetry breaking of the real complex is related to an energetic stabilization due to the helical distortion of the ligands, then a marginal stabilization of the *C*_2_ state that results from the Jahn–Teller effect that breaks the degeneracy of the pi bonding orbitals. These combined effects are ultimately what gives **Fe**_**3**_**L**_**3**_ its unique, high-spin ground state with short metal–metal bonds compared to previous EMACs and results in a *C*_2_ symmetric crystal structure. The near degeneracy of the *C*_2_ and *D*_3_ states and the similarity of their geometries is consistent with the solution phase NMR and UV/vis data that indicates a *D*_3_ symmetry for **Fe**_**3**_**L**_**3**_ at higher temperatures that relaxes to *C*_2_ in the low temperature crystal structure and Mössbauer data.

In addition to performing the high-symmetry analysis for **Fe**_**3**_**L′**_**3**_ to track down the origins of the symmetry breaking, the PES of the **Fe**_**3**_**L′**_**3**_ was also calculated and demonstrated to be relatively flat near the minimum energy with both semilocal DFT and RPA methods. This flat potential energy surface implies a range of geometric configurations could be realized in solution due to thermal fluctuations and solvent interactions, and this should be the case for **Fe**_**3**_**L**_**3**_ as well. Though multireference calculations indicate that the *S* = 6 and *S* = 5 spin-state manifolds overlap with one another, which likely has interesting implications for the low-temperature magnetic properties such as single-molecule magnetism, all methods predict a high-spin ground state in agreement with the room temperature magnetic data presently available. Strong ferromagnetic coupling was also predicted for nearest neighbor irons, while antiferromagnetic coupling was predicted between the outer irons in the linear chain. Overall these calculations demonstrate the ability of RPA to serve as a higher-level check on semilocal functionals and provide a more clear picture of the orbital ordering and PES in the **M**_**3**_**L**_**3**_ type EMACs made using a pyridine-type ligand that supports a high-spin ground state.

Given the small energy difference between *S* = 5 and *S* = 6 manifolds predicted by CASSCF, the finite orbital angular momentum of the sigma nonbonding configuration in *D*_3_ symmetry and the strong ferromagnetic coupling between nearest-neighbor irons predicted by RPA, the **Fe**_**3**_**L**_**3**_ molecule is a strong candidate for single molecule magnetism, and further experimentation is certainly warranted.

## Supporting Information Available

Additional computational details; results; and analyses (PDF)
Converged molecular structures and potential energy surfaces (ZIP)
