# Supplementary material for: Symmetry Breaking in a Triferrous Extended Metal Atom Chain
Source: Inorg Chem. 2024 Oct 10;63(42):19630–41. doi: 10.1021/acs.inorgchem.4c02752 (PMC11497207; doi:10.1021/acs.inorgchem.4c02752)
Supplement: Supplementary file 1 — ic4c02752_si_001.pdf [file ic4c02752_si_001.pdf]

Supporting Information

Symmetry breaking in a tri ferrous extended metal atom chain

Jefferson E. Bates[1]*, Jack N. McKeon[1], & Gary L. Guillet[2]
[1] Department of Chemistry & Fermentation Sciences, Appalachian State University, Boone NC
[2] Department of Chemistry, Furman University, Greenville SC
* Email address: batesje@appstate.edu

The Supporting Information is organized as follows. Additional computational details are presented, followed by the UV/Vis spectra results for $\mathbf{Fe_3L_3}$, results for the pi bonding configuration, $(\sigma)^2(\sigma_{nb})^1(\pi)^4$, and the impact of methyl groups on the molecular geometry. XYZ files for the optimized structures are gathered into zip files as part of the electronic supporting information. An explanation of the content of the zip file and its organization is included at the end.

Computational Details

For the DFT calculations reported herein, SCF energies and single-particle density matrices were converged to at least 10^{-7} a.u. and changes in the molecular Cartesian gradient norm were converged to 10^{-4} a.u. for structural optimizations. A fine quadrature grid (size m4 or larger) was used throughout. Resolution of the identity[1] and corresponding auxiliary basis sets[2,3] were used to accelerate the computation of the two-electron Coulomb repulsion integrals. A large frequency grid (at least 80 points) was used to calculate the RPA correlation energy[4], ensuring a sensitivity measure less than 10^{-5} a.u. for all species. 30 “levels” were used in the contour plots of the 2D potential energy surfaces. Analytical force constant[5] calculations were performed to check for saddle points or potential energy minima. For r^2SCAN and RPA analytical implementation of force constant calculations are not presently available in Turbomole.

While single-reference correlation methods are typically built on a Hartree-Fock reference state, RPA calculations in a DFT framework come with the additional complication of having to choose a reference semi-local potential to generate the occupied and virtual orbitals that are the inputs to an RPA calculation. This leads to variations in the results, especially as the fraction of hybrid mixing increases in the reference functional.[6] However, pure (semilocal) DFT functionals or hybrids with a small fraction of exchange are typically preferred over functionals with a large fraction of exact exchange. In order to distinguish these variations of RPA calculations, the semi-local functional is referred to as, for example, RPA@TPSS which indicates that the RPA results were evaluated using the TPSS reference determinant and Kohn-Sham potential. Using a Hartree-Fock reference for RPA calculations can lead to poor results[7] and is not recommended for transition metal chemistry without caution. Slower convergence compared to

semi-local functionals also requires RPA calculations to be checked with larger basis sets to ensure convergence.[8]

For the potential energy surfaces (PES) reported in the manuscript, a regular, two-dimensional grid of points was constructed using Fe–Fe distances between 2.1494 Å and 2.7494 Å, with a step size of 0.1 Å. For a given point on the PES, the iron atoms were held fixed while all other atoms were relaxed during the geometry optimization. A secondary grid was also constructed from 2.3994 to 2.5994 Å with a step size of 0.1 Å in order to provide more sampling around the potential energy minimum. This secondary grid was only utilized with a few functionals as it did not greatly impact the results. The unrelaxed RPA single-point energies were calculated assuming a frozen core to reduce the computational effort.

Theoretical, zero temperature gas-phase UV-visible spectra were generated from time dependent density functional theory (TDDFT) excitation energy calculations.[9] The optimized D_3 and C_2 structures for **Fe_3L_3** obtained from TPSSh with def2-SVP/TZVP basis sets were used for these simulations. The excitation energy calculations were carried out using TZVP basis sets for all atoms. Since the experimental spectra was measured in THF ($\epsilon = 7.52$), the impact of including solvation in the calculations through a continuum solvent model will likely be modest and was not pursued. A Gaussian broadening of 0.3 eV was used for each excitation to produce the smooth spectra reported in the manuscript. The excitation energies, oscillator strengths, and dominant orbital contributions are reported in Table S1 and S2 for selected transitions between 500 and 800 nm.

Mössbauer isomer shifts[10] (IS) and magnetic coupling constants[11] were calculated according to the procedures outlined in the literature. For both properties, TPSSh was used in conjunction with def2-TZVP basis sets for all atoms. For the *J*-couplings, RPA@TPSSh was also evaluated. Since RPA@TPSSh is evaluated in a "post-Kohn-Sham" manner, the expectation value for $\langle S^2 \rangle$ is equivalent to the value obtained in the self-consistent TPSSh calculations. Therefore to evaluate the RPA coupling constants, the total energy within the frozen core approximation was computed for each broken symmetry state, and the TPSSh value for $\langle S^2 \rangle$ of that state was used to finish calculating J_{12} and J_{23}. For the Mössbauer IS in D_3 symmetry for **Fe_3L_3**, the optimized geometry was calculated using def2-SVP/TZVP basis sets as described in the manuscript, and then a full TZVP single point energy calculation was used to determine the IS. For the C_2 IS of **Fe_3L_3**, the optimized structure obtained with TPSSh and TZVP basis sets for all atoms was used. For **$Fe_3L'_3$**, the optimized structure using TZVP basis sets for all atoms was used to calculate the IS in both D_3 and C_2 symmetry.

Fe_3L_3 UV/Vis Spectrum Analysis

The gas phase UV/Vis spectrum simulated for **Fe_3L_3** exhibits a number of excitations that originate from the amine nitrogen lone pair or occupied metal *d* orbitals and terminate in unoccupied metal *d* orbitals between 350 and 800 nm. The excitations predicted between 350 and 500 nm for D_3 and C_2 point groups are very similar, and therefore the focus of the analysis is on excitations between 500 and 800 nm. Table S1 and S2 contains a breakdown of the

occupied and virtual orbitals involved in the most intense transitions used to simulate the spectrum.

For D_3, Table S1, ~5 transitions are dominant in the 500 to 800 nm region. The excitation at 762 nm corresponds to a transition chiefly from the metals' sigma bonding (44 a1) and nonbonding (41 a2) orbitals to the unoccupied pi bonding orbital (76 e). The transition at 716 nm originates from an amine *p* type orbital on the L^{2-} ligand (40 a2) and terminates in the same metal pi bonding orbital (76 e), as do the two transitions around 650 nm. The transition at 567 nm is dominated by ligand *p* to metal *d*, delta-like orbital contributions. For the C_2 spectrum, Table S2, many more peaks appear with small oscillator strength due to the lower symmetry. Dominant transitions now appear at ~670 and ~510 nm, with other excitations of small, but non-negligible, oscillator strength in between. The overall character of these transitions matches that for the D_3 spectrum, though the intensities are noticeably different and the excitation energies are shifted to shorter wavelengths (higher energy). At room temperature in solution there is enough thermal energy to populate both ground states, so it is likely that the molecule is sampling configurations that coincide with each configuration, but on average resemble the D_3 geometry. The ^{1}H NMR coincides with a D_3 symmetric molecule which supports this idea.

Table S1 : Selected TDDFT excitations predicted with TPSSh using TZVP basis sets for the D_3 symmetric model of $\mathbf{Fe_3L_3}$. Oscillator strengths are reported as Osc str in the length gauge. Only contributions above 10% are reported for a given excitation.

Excitation	λ (nm)	Osc str (a.u.)	Occ Orbitals	Virt Orbitals	% weight
			44 a1	76 e	52.4
5 e	787	1.99E-04	41 a2	77 e	18.6
			40 a2	76 e	15.4
			41 a2	76 e	14.0
			44 a1	76 e	13.6
			41 a2	80 e	11.1
6 e	762	1.93E-03	41 a2	79 e	10.4
			40 a2	76 e	72.7
7 e	714	6.68E-03	45 a1	77 e	16.7
			74 e	76 e	59.1
9 e	649	5.10E-03	45 a1	77 e	22.0
2 a2	646	9.00E-03	74 e	76 e	94
			75 e	77 e	84.4
3 a2	567	5.98E-03	42 a2	46 a1	7.5

Table S2 : Selected TDDFT excitations predicted with TPSSh using TZVP basis sets for the C_2 symmetric model of $\mathbf{Fe_3L_3}$. Oscillator strengths are reported as Osc str in the length gauge. Occupied (occ) and virtual (virt) orbital contributions to each excitation are also reported. Only contributions above 10% are reported for a given excitation.

Excitation	λ (nm)	Osc str (a.u.)	Occ Orbitals	Virt Orbitals	% weight
4 a	665	3.56E-03	120 a	121 a	75.7
			120 a	122 a	48.8
			120 a	121 a	13.4
6 a	629	2.52E-03	116 b	121 b	10.4
10 a	567	4.61E-03	119 a	121 a	82.0
			120 a	118 b	21.2
			115 b	121 a	18.0
10 b	574	2.67E-03	116 b	125 a	15.6
			117 b	124 a	39.3
			117 b	123 a	13.3
12 b	542	2.60E-03	117 b	126 a	10.7
			117 b	125 a	53.3
15 b	514	2.79E-03	117 b	126 a	19.4

Alternative Pi Bonding Electron Configuration - $(\sigma)^2(\sigma_{nb})^1(\pi)^4$

While the main electronic configuration discussed in this work can be summarized as $(\sigma)^2(\sigma_{nb})^2(\pi)^3$, an alternative configuration was obtained in D_{3h} and D_3 symmetries that doubly occupied the beta-spin pi bonding orbitals and depopulated the sigma non-bonding orbital, $(\sigma)^2(\sigma_{nb})^1(\pi)^4$. This alternative configuration is expected to have a higher bond order due to the increased occupation of bonding orbitals which is confirmed by the optimized Fe–Fe bond lengths. Compared to the ground state configuration of $(\sigma)^2(\sigma_{nb})^2(\pi)^3$, this alternative configuration led to contractions of the Fe–Fe bond lengths by ~0.1 Å, Table S3, and was typically ~2-14 kcal/mol higher in energy, with hybrid functionals tending to predict a larger destabilization than semilocal functionals with increasing amounts of exact exchange. Reduction of symmetry from D_{3h} to D_3 introduced the torsion of the pyridine ligands seen previously, however the dihedral angle along the ligands is ~20° which is a few degrees less than that seen for the optimized structures in the $(\sigma)^2(\sigma_{nb})^2(\pi)^3$ occupation. Given the dissimilarity between the optimized Fe–Fe bond lengths obtained with this alternative state and the crystal structure, as well as the energetic destabilization, further calculations for this state were not explored.

Table S3: Optimized structural parameters for a suite of semi-local and hybrid functionals for the $(\sigma)^2(\sigma_{nb})^1(\pi)^4$ configuration. The significantly shortened Fe–Fe bonds are a direct result of the population of two pi bonding orbitals instead of a mixed pi bonding and sigma nonbonding occupation for the beta-spin electrons. The energy difference between the $(\sigma)^2(\sigma_{nb})^1(\pi)^4$ and $(\sigma)^2(\sigma_{nb})^2(\pi)^3$ configurations is also reported for each functional.

**$Fe_3L'_3$**	d(Fe–Fe) (Å)	d(Fe–N) (Å)	pyridine dihedral	Energy vs $(\sigma)^2(\sigma_{nb})^2(\pi)^3$ (kcal/mol)
BP	2.341	1.987	20.6	1.9
TPSS	2.330	1.986	21.2	4.9
r^2SCAN	2.318	1.980	20.1	9.7
TPSSh	2.309	1.986	20.7	10.7
B3LYP	2.290	2.004	20.0	13.6

In addition to the structural differences, Mössbauer isomer shifts were calculated for the D_3 TPSSh structure optimized with TZVP basis sets.[10] The isomer shifts predicted for the pi bonding configuration are 0.47 and 0.37 mm s^{-1} for Fe_1 and $Fe_{2,3}$, respectively, which differ from the experimentally observed values of 0.52 and 0.46 mm s^{-1}, as well as from the values calculated for the sigma nonbonding configuration in D_3 symmetry, 0.51 and 0.40 mm s^{-1}. This is another piece of evidence that supports the assignment of the S = 6 ground state to the sigma nonbonding configuration.

Addition of methyl groups to **$Fe_3L'_3$** and the impact on dihedral angles

In order to study the impact of adding additional methyl groups to the simplified model, **$Fe_3L'_3$**, the BP functional was utilized in combination with the def2-SVP/TZVP mixed basis sets described in the manuscript. One, two, and three methyl groups were successively added to interpolate between **$Fe_3L'_3$** (proteo form) and the full **Fe_3L_3** complex. After identifying the lowest energy positions for substitution for $–SiH_2Me$ (L'^1) and $–SiHMe_2$ (L'^2), geometry optimizations were performed for the ideal D_{3h} structures. After performing a frequency analysis, all of the obtained structures were saddle points with vibrations that contort the ligands. Lowering the symmetry of these compounds and distorting along the imaginary vibrational modes produced structures with lower symmetry such as D_3, C_3, or C_2. These structures all developed torsion angles along the L' ligand to varying degrees due to the interactions between methyl substituents on neighboring silyl groups and the benzene backbone of the ligand, Table S4. A single methyl substitution does not make a noticeable difference on the $N–Fe_3–Fe_2–N$ torsion angle, while introducing two increases this angle by ~4 degrees. Addition of the third methyl group is what ultimately leads to the steric crowding and the ~45 degree dihedral angle along

the ligand. The Fe–Fe bond lengths remain close to their values reported in Tables 1 and 2 of the manuscript.

Table S4: Average N–Fe_3–Fe_2–N dihedral angles for different model compounds with increasing number of methyl substitutions on the silyl groups of L^{2-} in D_3 symmetry. As the number of methyl groups is increased, the dihedral angle does not change much at first, but increases marginally from 1 to 2 and then again substantially from 2 to 3 methyl groups. This increase is due to the steric bulk of the methyl groups minimizing interactions with one another and with nearby atoms on the benzene rings of L^{2-}.

**BP@SVP/TZVP**	d(Fe–Fe) (Å)	pyridine dihedral
**$Fe_3L'_3$**	2.451	25.2
**$Fe_3(L'^1)_3$**	2.452	24.9
**$Fe_3(L'^2)_3$**	2.457	28.6
**Fe_3L_3**	2.411	43.7

The interactions being minimized are illustrated in Figure S1 for **Fe_3L_3**. By adopting a large dihedral angle, there is more room for the silyl groups to rotate. As a result, the distances between the inner methyl groups increases (left), as well as the distance between hydrogens on the rotated methyl group and the hydrogen on the benzene ring (right).

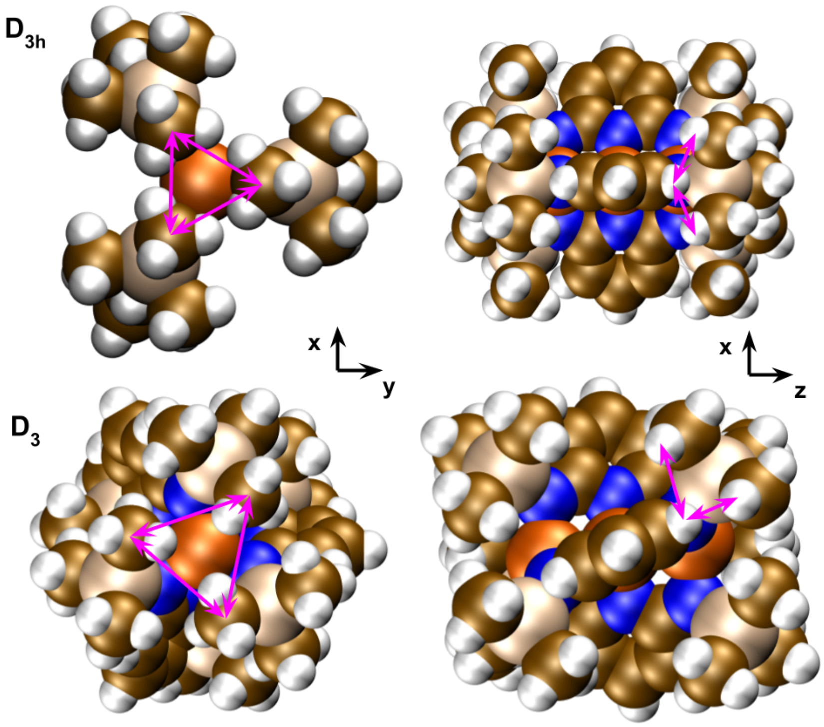

Figure S1: Space-filled axial (left) and equatorial (right) views for **Fe_3L_3** in D_{3h} (top) and D_3 (bottom) symmetry. These structures were optimized using TPSSh with mixed SVP/TZVP basis sets. The inner methyl groups are in close proximity in D_{3h}, as are the silyl groups to the left and

right of the benzene rings. By making a torsion along the ligand, and rotating around silicon, these groups minimize their interactions and produce the large torsion angle seen in the experimental crystal structure.

CASSCF Results for **$Fe_3L'_3$**

Figure S2 contains the converged active space orbitals obtained using the SVP/TZVP basis sets and (18,15) active space with 29 states included in the state-average. The similarity between the CAS and DFT orbitals for the occupied sigma and pi orbitals is striking. The dominant configuration of the ground state matches the occupation predicted by DFT for the full **Fe_3L_3** complex, further supporting the applicability of the model **$Fe_3L'_3$** in mimicking the important features of the real complex. The central iron atom does not appear to be coupled to the other two irons via their delta orbitals, and instead the metal-metal interaction arises from the sigma and pi orbitals.

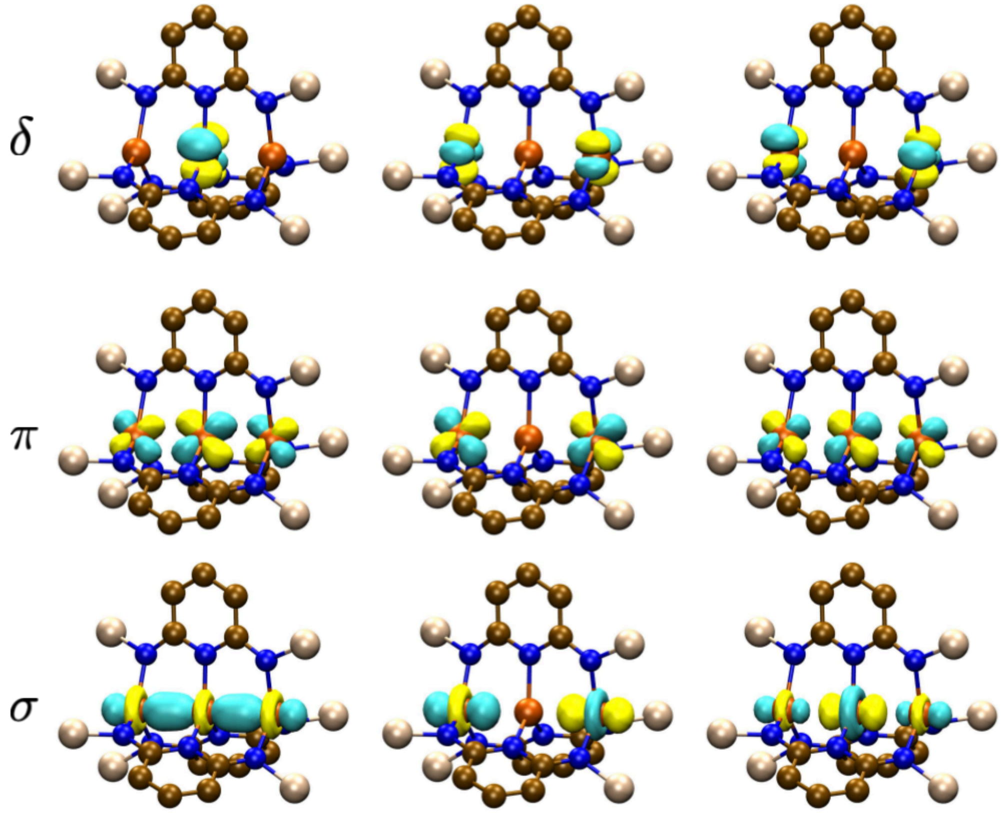

Figure S2: Contour plots of the converged (18,15) CASSCF active space orbitals calculated using 29 states of the S = 6 manifold at the C_2 geometry obtained with BP SVP/TZVP basis sets. A contour value of 0.05 was used for all of these plots, and hydrogen atoms have been suppressed for clarity. For the pi and delta orbitals, only half of the orbitals are shown as the other 6 can be obtained by 90 degree rotations.

Description of compressed files with structures

The structure files and potential energy surfaces (PES) contained in the compressed file correspond to the optimized structures described in the main publication. A summary of these additional files can be found below.

1. fe3lp3_rirpa_structures.zip : this file contains the def2-SVP/TZVP optimized RPA structures obtained in C_1 symmetry for different reference semilocal functionals for the **$Fe_3L'_3$** compound. The semilocal reference functional is indicated at the end of the file name.
2. fe3l3_dft_structures.zip : this file contains the optimized structures for **Fe_3L_3** obtained using def2-TZVP basis sets for all atoms with TPSS, TPSSh, and RPA.
3. fe3lp3_dft_structures.zip : this file contains the optimized structures for **$Fe_3L'_3$** obtained using def2-TZVP basis sets for all atoms with BP86, TPSS, r^2SCAN, TPSSh, and B3LYP. The point group symmetry is indicated in the file name along with the functional. In addition to the $(\sigma)^2(\sigma_{nb})^2(\pi)^3$ results (indicated as sigma_nonbond), the optimized structures for the $(\sigma)^2(\sigma_{nb})^1(\pi)^4$ occupation are also reported (indicated as pi_occ).
4. 2d-b3lyp-tzvp.zip : this file contains the optimized structures corresponding to the 2D PES calculated with the B3LYP functional using def2-TZVP basis sets for all atoms. These structures were used as input to calculate the RPA@B3LYP PES.
5. 2d-tpss-tzvp.zip : this file contains the optimized structures corresponding to the 2D PES calculated with the TPSS functional using def2-TZVP basis sets for all atoms. These structures were used as input to calculate the RPA@TPSS PES. Note that the secondary grid structures are also included in this file.
6. 2d-pes-tzvp_combined.csv : this file contains the 2D PES data for TPSS, RPA@TPSS, B3LYP, and RPA@B3LYP obtained with def2-TZVP basis sets for all atoms. This comma separated value (CSV) file is organized by the different Fe–Fe bond lengths (Å).

References

(1) Vahtras, O.; Almlöf, J.; Feyereisen, M. W. Integral Approximations for LCAO-SCF Calculations. *Chem. Phys. Lett.* **1993**, *213* (5), 514–518. https://doi.org/10.1016/0009-2614(93)89151-7.
(2) Weigend, F.; Ahlrichs, R. Balanced Basis Sets of Split Valence, Triple Zeta Valence and Quadruple Zeta Valence Quality for H to Rn: Design and Assessment of Accuracy. *Phys. Chem. Chem. Phys.* **2005**, *7* (18), 3297–3305. https://doi.org/10.1039/B508541A.
(3) Weigend, F. Accurate Coulomb-Fitting Basis Sets for H to Rn. *Phys. Chem. Chem. Phys.* **2006**, *8* (9), 1057–1065. https://doi.org/10.1039/B515623H.
(4) Eshuis, H.; Yarkony, J.; Furche, F. Fast Computation of Molecular Random Phase Approximation Correlation Energies Using Resolution of the Identity and Imaginary Frequency Integration. *J. Chem. Phys.* **2010**, *132* (23), 234114. https://doi.org/10.1063/1.3442749.
(5) Deglmann, P.; Furche, F.; Ahlrichs, R. An Efficient Implementation of Second Analytical Derivatives for Density Functional Methods. *Chem. Phys. Lett.* **2002**, *362* (5), 511–518. https://doi.org/10.1016/S0009-2614(02)01084-9.
(6) Bates, J. E.; Mezei, P. D.; Csonka, G. I.; Sun, J.; Ruzsinszky, A. Reference Determinant Dependence of the Random Phase Approximation in 3d Transition Metal Chemistry. *J.*

Chem. Theory Comput. **2017**, *13*, 100–109. https://doi.org/10.1021/acs.jctc.6b0090.
(7) Rekkedal, J.; Coriani, S.; Iozzi, M. F.; Teale, A. M.; Helgaker, T.; Pedersen, T. B. Communication: Analytic Gradients in the Random-Phase Approximation. *J. Chem. Phys.* **2013**, *139* (8), 081101. https://doi.org/10.1063/1.4819399.
(8) Eshuis, H.; Furche, F. Basis Set Convergence of Molecular Correlation Energy Differences within the Random Phase Approximation. *J. Chem. Phys.* **2012**, *136* (8), 084105. https://doi.org/10.1063/1.3687005.
(9) Bates, J. E.; Furche, F. Harnessing the Meta-Generalized Gradient Approximation for Time-Dependent Density Functional Theory. *J. Chem. Phys.* **2012**, *137*, 164105.
(10) Römelt, M.; Ye, S.; Neese, F. Calibration of Modern Density Functional Theory Methods for the Prediction of 57Fe Mössbauer Isomer Shifts: Meta-GGA and Double-Hybrid Functionals. *Inorg. Chem.* **2009**, *48* (3), 784–785. https://doi.org/10.1021/ic801535v.
(11) Szarek, P.; Wegner, W.; Grochala, W. Ferromagnetic Ground State for a Hypothetical Iron-Based Extended Metal Atom Chain. *J. Mol. Model.* **2016**, *22* (3), 63. https://doi.org/10.1007/s00894-016-2928-x.
